# Impact of Intravenous Trehalose Administration in Patients with Niemann–Pick Disease Types A and B

**DOI:** 10.3390/jcm11010247

**Published:** 2022-01-04

**Authors:** Moein Mobini, Shabnam Radbakhsh, Francyne Kubaski, Peyman Eshraghi, Saba Vakili, Rahim Vakili, Manijeh Khalili, Majid Varesvazirian, Tannaz Jamialahmadi, Seyed Ali Alamdaran, Seyed Javad Sayedi, Omid Rajabi, Seyed Ahmad Emami, Željko Reiner, Amirhossein Sebkar

**Affiliations:** 1 Faculty of Medicine, Mashhad University of Medical Sciences, Mashhad 9177948564, Iran; Mobinim891@gmail.com; 2 Student Research Committee, Mashhad University of Medical Sciences, Mashhad 9177948564, Iran; Radbakhshs971@mums.ac.ir; 3 Department of Medical Biotechnology and Nanotechnology, Mashhad University of Medical Sciences, Mashhad 9177948564, Iran; 4 Department of Genetics, UFRGS, Porto Alegre 91501970, Brazil; fkubaski@udel.edu; 5 Medical Genetics Service, HCPA, Porto Alegre 90035903, Brazil; 6 Biodiscovery Lab, HCPA, Porto Alegre 90035903, Brazil; 7 Department of Pediatric Diseases, Akbar Hospital, Faculty of Medicine, Mashhad University of Medical Sciences, Mashhad 9177897157, Iran; Eshraghip2@mums.ac.ir; 8 Medical Genetic Research Center, Mashhad University of Medical Sciences, Mashhad 9177948564, Iran; Vakilis@mums.ac.ir (S.V.); Vakilir@mums.ac.ir (R.V.); 9 Children and Adolescents Health Research Center, Research Institute of cellular and Molecular Science in Infectious Diseases, Zahedan University of Medical Science, Zahedan 9816743463, Iran; dr_khalili2000@yahoo.com; 10 Shafa Hospital, Kerman University of Medical Sciences, Kerman 7618751151, Iran; dr.vazirian@gmail.com; 11 Department of Nutrition, Faculty of Medicine, Mashhad University of Medical Sciences, Mashhad 9177948564, Iran; jamiat931@gmail.com; 12 Pediatric Radiology Department, Faculty of Medicine, Mashhad University of Medical Sciences, Mashhad 9177948564, Iran; Alamdarana@mums.ac.ir; 13 Department of Pediatrics, Mashhad University of Medical Sciences, Mashhad 9177948564, Iran; Sayedij@mums.ac.ir; 14 Department of Pharmaceutical and Food Control, School of Pharmacy, Mashhad University of Medical Sciences, Mashhad 9177948954, Iran; Rajabio@mums.ac.ir; 15 Department of Traditional Pharmacy, School of Pharmacy, Mashhad University of Medical Sciences, Mashhad 9177948954, Iran; Emamia@mums.ac.ir; 16 Department of Internal Medicine, University Hospital Center Zagreb, University of Zagreb, Kišpatićeva 12, 1000 Zagreb, Croatia; zreiner@kbc-zagreb.hr; 17 Applied Biomedical Research Center, Mashhad University of Medical Sciences, Mashhad 9177948564, Iran; 18 Biotechnology Research Center, Pharmaceutical Technology Institute, Mashhad University of Medical Sciences, Mashhad 9177948954, Iran; 19 Department of Biotechnology, School of Pharmacy, Mashhad University of Medical Sciences, Mashhad 9177948954, Iran

**Keywords:** lysosomal storage disease (LSD), Niemann–Pick type A, Niemann–Pick type B, acid sphingomyelinase, sphingolipid deposition, trehalose

## Abstract

Background and Aims: Niemann–Pick disease (NPD) types A (NPA) and B (NPB) are caused by deficiency of the acid sphingomyelinase enzyme, which is encoded by the *SMPD1* gene, resulting in progressive pathogenic accumulation of lipids in tissues. Trehalose has been suggested as an autophagy inducer with therapeutic neuroprotective effects. We performed a single-arm, open-label pilot study to assess the potential efficacy of trehalose treatment in patients with NPA and NPB patients. Methods: Five patients with NPD type A and B were enrolled in an open-label, single-arm clinical trial. Trehalose was administrated intravenously (IV) (15 g/week) for three months. The efficacy of trehalose in the management of clinical symptoms was evaluated in patients by assessing the quality of life, serum biomarkers, and high-resolution computed tomography (HRCT) of the lungs at the baseline and end of the interventional trial (day 0 and week 12). Results: The mean of TNO-AZL Preschool children Quality of Life (TAPQOL) scores increased in all patients after intervention at W12 compared to the baseline W0, although the difference was not statistically significant. The serum levels of lyso-SM-509 and lyso-SM were decreased in three and four patients out of five, respectively, compared with baseline. Elevated ALT and AST levels were decreased in all patients after 12 weeks of treatment; however, changes were not statistically significant. Pro-oxidant antioxidant balance (PAB) was also decreased and glutathione peroxidase (GPX) activity was increased in serum of patients at the end of the study. Imaging studies of spleen and lung HRCT showed improvement of symptoms in two patients. Conclusions: Positive trends in health-related quality of life (HRQoL), serum biomarkers, and organomegaly were observed after 3 months of treatment with trehalose in patients with NPA and NPB. Although not statistically significant, due to the small number of patients enrolled, these results are encouraging and should be further explored.

## 1. Introduction

Niemann–Pick disease (NPD) is a lysosomal storage disorder (LSDs) caused by the deficiency of acid sphingomyelinase activity (ASM) NP type A and B or cholesterol transporter function (NP type C) leading to lipid accumulation in different tissues and organs [1]. The estimated prevalence of NPA and NPB is 0.4–0.6 in 100,000 individuals [2]. Hepatosplenomegaly, pulmonary insufficiency, and profound central nervous system (CNS) involvement can lead to death in untreated patients within the first few years of life in NPA [3]. In contrast, NPB is the non-neuropathic form of the disorder with milder symptoms and clinical manifestations starting at later ages, with most patients reaching adulthood [4]. Low levels or total deficiency of ASM is the main cause of sphingomyelin accumulation and lipid abnormalities as well as downstream cell signaling pathways that affect ceramide generation as an important secondary pathway [1]. A common histopathological occurrence in NP patients is lipid-laden macrophages, also called foam cells, in the liver, spleen, lung airways, bone marrow, and cerebral cortex that lead to progressive destruction of target tissues [5]. Early diagnosis and treatment are required to attenuate outcome and to improve the quality of life in NP patients; however, bone marrow transplantation (BMT), enzyme replacement therapy (ERT), and other therapeutic approaches are still in stages of research and have not been adequately effective [6,7,8].

Trehalose is a natural non reducing (1–1 α-linkage) disaccharide in various organisms, from bacteria to animals, that exerts cell-protective effects under tensions, such as temperature, drought, and oxidative stress [9]. Trehalose has been recognized as a safe additive by the Joint WHO/FOA Expert Committee on Food Additive (JECFA) and U.S. Food and Drug Administration (FDA) in 2000, and was approved for use in food in Europe in 2001 [10]. Apart from basic and experimental evidence [11,12,13,14,15,16], several clinical trials were performed to evaluate the safety and efficacy of trehalose in healthy subjects or patients with different diseases, both orally and intravenously [17,18]. At doses up to 50 g, trehalose is safe for humans, and no adverse effect has been reported in most subjects; however, gastrointestinal side effects may occur in trehalose-deficient individuals [19].

In addition, trehalose has also been reported to prevent neuronal damage and attenuate neurodegenerative disorders caused by LSDs [19,20]. Antiaggregant, anti-inflammatory, and antioxidant properties, along with autophagy inducer, might be proposed as potential mechanisms of neuroprotective activities of trehalose in both cell cultures and in-vivo animal models [21,22]. Several lines of evidence suggest the chaperone-like activity of trehalose to prevent protein misfolding or aggregation and to contribute to clearance of accumulated proteins through promoting autophagy in neurodegenerative diseases (NDs) [21]. As such, trehalose is emerging as a novel therapeutic alternative to repressing oxidative stress and inflammation by decreasing the production of reactive oxygen species (ROS) and proinflammatory cytokines, such as interleukin 1 beta (IL-1β) and tumor necrosis factor-alpha (TNF-α), respectively [23]. Deposition of sphingomyelin and other lipids [24], neuroinflammation [25], and oxidative stress [26] have been considered as leading causes of NP. Therefore, trehalose might be effective at attenuating the negative outcomes in NP patients by reducing lipid accumulation, inflammation, and oxidative damage [9,27]. Trehalose can be used by either oral or intravenous (IV) administration; however, its absorption is decreased to 0.5% in the oral route due to enzymatic metabolization with Trehalase exhibiting in the intestinal brush border, and (IV) trehalose administration is more efficient for clinical trials [10,28]. Nevertheless, oral administration of trehalose in both preclinical and clinical studies of oculopharyngeal muscular dystrophy (OPMD) and Machado–Joseph disease (MJD) can stabilize neurological impairment and improve the severity of clinical disease scores [17,18].

This study reports clinical research aimed to investigate the efficacy of intravenous trehalose infusion (15 g/week) for a period of 12 weeks in five NPA and NPB patients.

## 2. Patients and Methods

### 2.1. Study Design and Participants

A single-arm, open-label pilot study was performed to assess trehalose therapeutic potential in NPA and NPB patients. All patients received IV trehalose infusions once a week (15 gr) for 90 min during three months of treatment. Follow-up visits were also conducted weekly during the study period. This clinical research was approved by the Ethics Committee of the Mashhad University of Medical Sciences, registered in the Iranian Registry of Clinical Trials (Code: IRCT20130829014521N16). Five patients aged 2–12 years old who had been diagnosed with NPA and NPB (confirmation by genotype and clinical examination) were considered for enrollment in the present study. The parents or legal guardians of the children signed the informed consent forms before any procedures were performed.

### 2.2. Test Substances

For our research, the pharmaceutical grade of trehalose has been used as a form of aqueous 15% solution in 100 mL sterile sealed vials manufactured by Dr. Rajabi Pharmaceutical Company, Khorasan Razavi, Iran.

### 2.3. Endpoints and Assessments

The main objective was to determine the therapeutic efficacy of trehalose in patients with NPA and NPB. Primary endpoints included quality of life assessment and reduction in serum biomarker levels (lysosphingomyelin-lysoSM, and lysosphingomyelin-509 (lysoSM-509)). The secondary endpoints of the study were to assess the condition of the liver, spleen, and lung, and measurement of the aminotransferases enzymes (AST and ALT levels), as well as oxidative stress status at the baseline and end of the interventional trial (day 0 and week 12).

#### 2.3.1. Primary Endpoints

Quality of life assessment: TAPQOL (TNO-AZL Preschool children Quality of Life) index was used during this research to evaluate the physical, social, emotional, and cognitive function of patients. TAPQOL is a multidimensional questionnaire-parent form with 43 items comprising 12 scales, which was developed to measure health-related quality of life (HRQoL) in preschool children (aged 2–48 months) [29].

Sample preparation and lyso-SM and lyso-SM-509 quantification: Blood samples were obtained from all patients before and after treatment. Samples were collected in tubes containing serum gel separator and were centrifuged at 750× *g* for 20 min to obtain serum. Serum samples were aliquoted and were stored at −80 °C until required for measurements. Changes in serum lyso-SM and lyso-SM-509 levels were measured by ultra-performance liquid chromatography tandem mass spectrometry (UPLC–MS/MS) in a Xevo TQ-S micro (Waters Technology, Milford, MA, USA) at baseline (day 0) and the end of the study (week 12). The method used for the quantification of lyso-SM and lyso-SM-509 was adapted from Polo et al., 2019 [30].

#### 2.3.2. Secondary Endpoints

Liver, spleen, and lung scans: Serum levels of alanine aminotransferase (ALT) and aspartate transaminase (AST) were measured by the kinetic method using a colorimetric assay kit to assess liver function at baseline and the end of the trehalose treatment period. Moreover, the spleen and liver size were measured using ultrasonography, and volumetric analyses were performed at the baseline and end of the study. Chest high-resolution computed tomography (HRCT) was also performed to compare the lung condition of patients between the W0 and W12.

Oxidative stress status: To evaluate whether trehalose could improve the antioxidant status, investigation of (anti)oxidant parameters pre- and post-treatment were performed by commercial kits (Kiazist; Iran). In this study, PAB (pro-oxidant antioxidant balance) was measured to evaluate the total oxidants and antioxidants in a single measurement simultaneously according to the previously described method [31], which is based on the oxidation of the chromogen 3,3′,5,5′-tetramethylbenzidine (TMB) to a color cation by pro-oxidants in an enzymatic reaction and reduction of the TMB cation to a colorless compound in a chemical reaction. The antioxidant enzyme activity of the glutathione peroxidase (GPx) was also assayed based on the reduction of hydrogen peroxide to water accompanied by the oxidation of glutathione.

#### 2.3.3. Statistical Analysis

Statistical analysis was performed with GraphPad Prism version 8 software and Microsoft Excel (2019) The results were analyzed using paired *t*-test to evaluate the significance of differences before and after the treatment period. Results with *p* < 0.05 were considered statistically significant.

## 3. Results

### 3.1. Clinical Characteristics of Patients

Five male patients with a mean age of 4.4 years (range = 2–12 years of age) were enrolled who were diagnosed clinically and genetically with Niemann–Pick (NP) type A and B, genotype analysis results were homozygous. All children were born from consanguineous families. No subjects discontinued from the study, and all patients received all 12 of their scheduled doses.

### 3.2. Quality of Life Assessment

**Figure 1:**
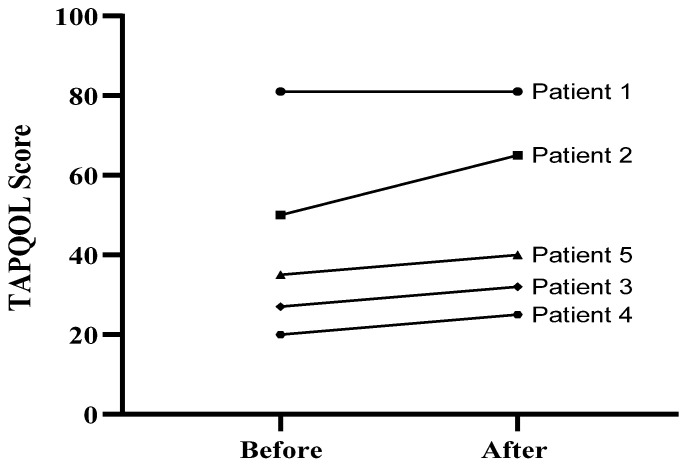
TAPQOL score of five patients at baseline (week 0) and at the end of treatment (week 12).

TAPQOL Test: To determine whether trehalose treatment could improve the health status in patients, we compared the TAPQOL score, which was used to assess the patients’ health-related quality of life between the W12 and W0. The TAPQOL index score can vary from 0–100, and higher scores indicate better quality of life. The TAPQOL score was elevated in all patients, and the mean score for quality of life was increased after intervention at W12 compared to the baseline (difference between means ± standard error mean (SEM): 6/000 ± 2/449), although the difference was not statistically significant. The results suggested an improvement in health-related quality of life after 12 weeks of trehalose treatment (Figure 1) in patients 2–5.

### 3.3. Serum Lysosphingomyelin Levels (lyso-SM and lyso-SM509)

**Figure 2:**
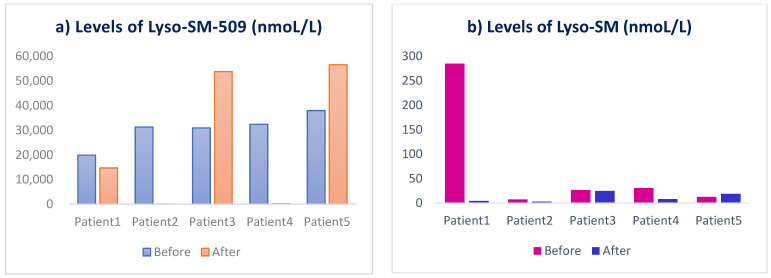
Levels of serum lysosphingomyelin (**a**) lyso-SM509 and (**b**) lyso-SM in patients before and after 3 months treatment with trehalose.

The levels of serum lysosphingomyelin are shown at baseline and week 12 (Figure 2). The average of lyso-SM-509 at the baseline was 30.511 (nmoL/L), while the average post-treatment is 25.051 (nmoL/L); the average of lyso-SM at the baseline was 72 (nmoL/L) while the average post-treatment was 12 (nmoL/L). Overall, out of five patients there was a reduction in the levels of lyso-SM-509 in three patients, and a reduction in levels of lyso-SM in four patients (Figure 2). However, the changes were not statistically significant.

### 3.4. Serum ALT and AST Levels

**Figure 3:**
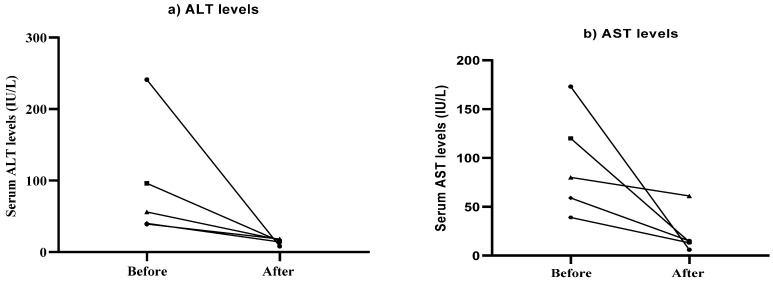
Alterations in serum ALT (**a**) and AST (**b**) levels in patients following 12 weeks of treatment with trehalose.

The average of ALT at the baseline was 94.40 (IU/L), while the average post-treatment was 14.40 (IU/L); the average of AST at the baseline was 94.20 (IU/L), while the average post-treatment was 21.80 (IU/L). Overall, there was a reduction in the levels of ALT and AST post-treatment (Figure 3). Although changes were not statistically significant, improved results (reduction in ALT and AST levels) showed improvement in liver function after trehalose treatment.

### 3.5. Oxidative Stress Index (OSI)

**Figure 4:**
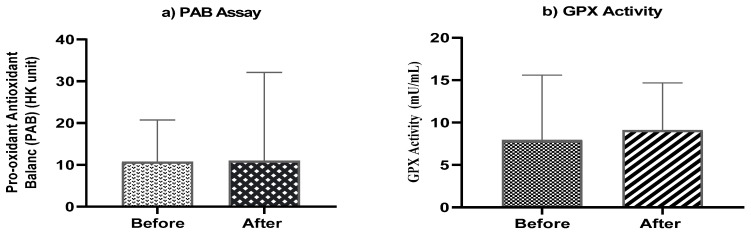
(**a**) Mean serum pro-oxidant antioxidant balance (PAB) and (**b**) mean serum activities of GPX (mU/mL) at baseline and end of the study.

The mean of pro-oxidant-antioxidant balance (PAB) before treatment was 10.734 (HK unit), while post-treatment was 11.018 (HK unit) (Figure 4b). The level of GPX activity before treatment was 7.93 (mU/mL), while post-treatment was 9.09 (mU/mL) (Figure 4a). Although, differences were not statistically significant neither in PAB values nor in GPX activity.

### 3.6. Sonographic Liver and Spleen Dimensions

Table 1 includes the alteration of spleen and liver size in patients pre-and post-treatment with trehalose. Although spleen size was found to have decreased in two patients (patients 2 and 4) compared with baseline, a progressive increase in the mean splenic length and the average liver volume was observed at the end of the study. It is worth mentioning that the liver diameter was reported by measuring the liver span below the costal margin in the midclavicular line by using the ultrasound scan because it could assist clinicians to confirm these changes in practice.

### 3.7. Lung HRCT

**Figure 5:**
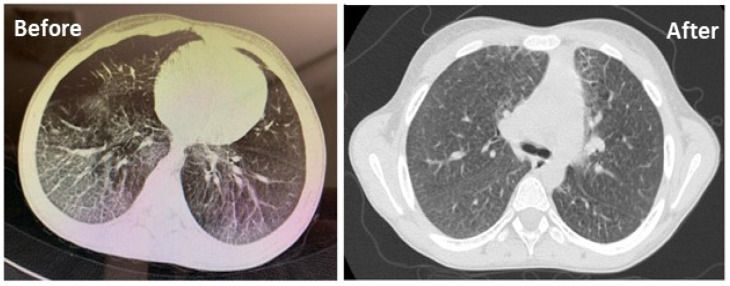
HRCT scan of the chest showing ground–glass changes in both lung fields before and after 3 months of treatment with trehalose in a patient.

Follow-up HRCT chest was carried out in all patients. Improvement of symptoms in HRCT chest findings were observed in two patients out of five after 3 months treatment with trehalose (Figure 5).

## 4. Discussion

ASMD (acid sphingomyelinase deficiency), also known as Niemann–Pick disease, is a rare autosomal recessive LSD that includes two subtypes (A and B) associated with lipid metabolism abnormalities and intracellular deposition of glycosphingolipids [32]. Abnormal lipid accumulation due to a deficiency of specific lysosomal enzymes has been shown to impact morphologic alterations in different tissues, leading to multi-organ failure and early death in children with NPA and NPB [3]. Currently, no effective treatment is available for NPA/NPB patients [33]. A considerable body of evidence suggests the role of impaired autophagy in the pathophysiology and progression of lipid storage disorders [34,35,36]. Therefore, the possible use of the autophagy-inducing compounds in decreasing lipid accumulation has been proposed to attenuate severe LSDs manifestations [37]. In recent years, trehalose has been described as a natural non-reducing disaccharide that promotes the autophagy process in both in vitro and in vivo models by activating transcription factor EB (TFEB) and enhancing target genes such as *GLA*, *LAMP2A*, *MCOLN1*, *CTSB*. Furthermore, it can also induce the autophagy process via the mTOR-independent pathway in cells of the nervous system [38,39,40,41]. This study aimed to evaluate the potential efficacy of IV trehalose (at a dose of 15 mg/week) in NPA and NPB patients. The dose of 15 mg/week was selected based on recent evidence and a previous similar clinical study showing the safety and efficacy of 15 mg IV trehalose in patients with Machado–Joseph disease (MJD) [18]. We hypothesized that trehalose could slow disease progression and improve neuropathologic features by decreasing sphingolipid deposition post three months of treatment. Disruption of sphingolipid homeostasis leads to several pathological consequences, and the accumulation of these metabolites can trigger a high level of apoptosis by activating proapoptotic genes and proteins [42,43]. Elevated levels of lysosphingolipids (lyso-SM and lyso-SM-509) have been identified as specific and reliable biomarkers for the diagnosis of NP and early assessment of drug effects during the treatment process in all types of NP (A/B and C), which might be detected via different methods in plasma, serum, or dried blood spots of patients [30,44]. The results of this study might suggest that treatment with trehalose could potentially lead to a decrease in the levels of both lyso-SM and lyso-SM-509; however, additional studies are required to further elucidate the efficacy of trehalose treatment and to confirm if the mechanism is associated with the autophagy-inducing effect of this small molecule that contributes to the clearance of accumulated lysosomal lipid substrates.

In addition, as important signaling mediators involved in the control of cell survival, sphingolipids also have an essential role in regulating proinflammatory cytokines and inflammation processes. Sphingosine-1-phosphate can induce interleukin 8 (IL-8) expression and activated protein 1 (AP-1) inflammatory transcriptional action via activating ERK and p38 MAPK pathways, which are involved in many inflammatory responses, particularly in lung inflammation and progressive respiratory failure [45,46]. Pulmonary involvement is considered one of the main causes of morbidity and mortality in NPA and NPB patients [47]. It has been shown that trehalose can attenuate inflammation in different animal models by reducing the production of inflammatory cytokines, such as TNF-α, MCP-1 (monocyte chemotactic protein-1) and PAI-1 (plasminogen activator inhibitor–1) [23]. Our lung function tests and high-resolution computed tomography (HRCT) findings showed improved lung function in two patients during three months of trehalose treatment that might be due to anti-inflammatory effects of trehalose and modulation of pro-inflammatory cytokines. Furthermore, recent studies have uncovered the link between sphingolipid deposition and cellular stress responses, such as ER and oxidative stress [48,49]. The accumulation of complex sphingolipid inositol phosphorylceramide (IPC) can increase ROS generation in mitochondria, which in turn decreases mitochondrial mass by activating Ras and affecting Snf1/AMPK pathways [49]. It has also been suggested that trehalose might exhibit a protective effect against oxidative stress by either upregulation of antioxidant enzymes such as superoxide dismutase (SOD), glutathione peroxidase (GPX) [50,51], or scavenging ROS [52]. In line with this, we investigated pro-oxidant antioxidant balance and GPX activity after treatment to evaluate if trehalose has antioxidant effects. A decrease in levels of PAB and increased GPX activity could be due to the antioxidant activity of trehalose in the serum of NPA and NPB patients.

Besides anti-inflammatory and antioxidant properties, neuroprotective effects of trehalose to ameliorate neurological pathologies have been established in several experimental models of neurodegenerative diseases (NDs) [19]. Significant improvement has been observed on multiple behavioral tasks along with a marked increase in synaptophysin, doublecortin, and progranulin in the hippocampus and cortex of mice treated with oral administration of 2% trehalose for one month [53,54,55]. Moreover, a clinical study showed the effect of trehalose in patients with MJD with the optimal dose of 15 mg/week to improve disease severity and clinical symptoms [18]. Neurological involvement in NP varies in frequency and severity of disease, loss of mental abilities, and cognitive impairment more prominent in NPA, while type B patients tend to have milder symptoms with later-onset [56]. Our data confirmed previous similar reports in the literature and demonstrated significant improvement in health-related quality of life assessment through increased TAPQOL scores in four out of five patients after three months of treatment.

Hepatosplenomegaly accompanied by liver failure is another typical sign in NP patients [56]. Two clinical studies indicated liver dysfunction and elevated transaminase levels (ALT and AST) in 51% to 75% of NP patients [57,58]. Our results showed improvements in liver transaminase levels, and a reduction in the levels of both ALT and AST were observed in all patients treated with trehalose. Furthermore, a slight decrease in the spleen dimensions were found in two patients.

Our study has several limitations, including it being an open-label pilot research with limited sample size. Larger controlled, blind studies are required to demonstrate whether trehalose is effective in NPA and NPB patients. The length of treatment in our research is also not long enough to evaluate trehalose’s effects on behavioral problems. Finally, future dose-ranging studies are needed to indicate the optimal therapeutic dose of trehalose.

In conclusion, the treatment of NPA and NPB in patients with 15 mg/week of trehalose may be effective to reduce serum levels of sphingomyelins and possibly improving disease symptoms caused by lipid accumulation, although large-scale randomized trials with longer follow-up are needed to confirm whether trehalose has clinical efficacy in patients with LSDs.

## Figures and Tables

**Table 1:** Spleen and liver diameter changes pre-and post-treatment with trehalose.

Patient ID	Spleen Cranio-Caudal Diameter (mm)		Liver Diameter Changes, Measuring the Liver Span below the Costal Margin by Ultrasound Scan (mm)	
	Before	After	Before	After
01	204	224	30	30
02	125	122	10	30
03	120	138	30	10
04	150	145	10	30
05	115	115	30	30

## Data Availability

Data are available from the corresponding author upon a reasonable request.
